# Efficacy of baricitinib in treatment of coexisting alopecia areata and lichen planopilaris

**DOI:** 10.1016/j.jdcr.2024.10.011

**Published:** 2024-10-30

**Authors:** Adaugo A. Sanyi, Abigail M. Smith, Tiffany T. Mayo

**Affiliations:** Department of Dermatology, University of Alabama at Birmingham, Birmingham, Alabama

**Keywords:** alopecia areata, baricitinib, Black skin, coexisting alopecias, lichen planopilaris

## Introduction

Alopecia areata (AA) is an inflammatory disorder characterized by non-scarring hair loss that typically presents as well-defined patches of alopecia. In severe cases, it can progress to total loss of scalp and body hair (alopecia totalis/universalis). Lichen planopilaris (LPP) is a rare variant of lichen planus that primarily affects the hair follicles, leading to cicatricial alopecia. LPP often presents with perifollicular erythema, follicular hyperkeratosis, and progressive permanent hair loss.^1^ Baricitinib is a Janus kinase inhibitor that is used as treatment reserved for extensive or recalcitrant AA.^2^ Recent studies suggest that baricitinib is also a therapeutic option for LPP that is refractory to conventional treatments.^3^ This case report highlights an interesting and challenging scenario involving the concurrent presence of 2 clinically distinct conditions, AA and LPP, in a Black or African American female. We present this case to demonstrate therapeutic efficacy in treating concurrent disease with baricitinib, including scarring alopecia. This case also brings awareness to the simultaneous occurrence of different alopecia types in Black or African American patients and the importance of judicious dermatoscopic and histopathologic assessment.

## Case report

A 41-year-old Black or African American female presented to the University of Alabama at Birmingham dermatology clinic as a new patient for new rapid areas of hair loss on the bilateral parietal scalp weeks prior to visit. She also reported a 3-year history of progressive alopecia on the crown despite the use of potent topical steroids and multiple rounds of intralesional corticosteroids. She denied any associated scalp pruritis or pain. Her prior medical history included Hashimoto’s disease, for which she was taking levothyroxine 200 mcg per os daily. Additionally, she had a history of diabetes and was taking long-acting insulin and empagliflozin 25 mg PO daily at the time of visit.

**Fig 1:**
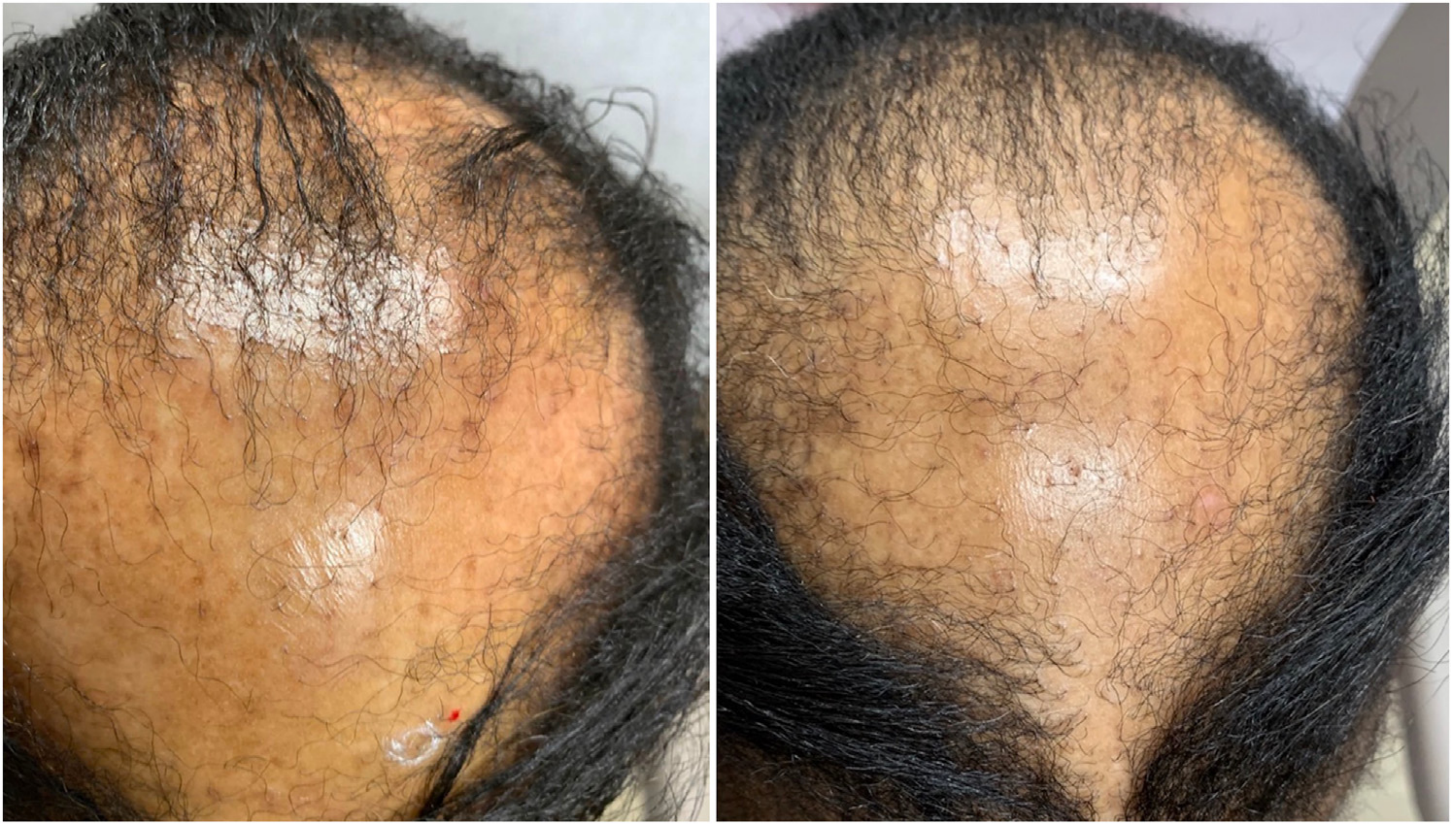
Lichen planopilaris before (*left*) and after (*right*) ) treatment with baricitinib.

**Fig 2:**
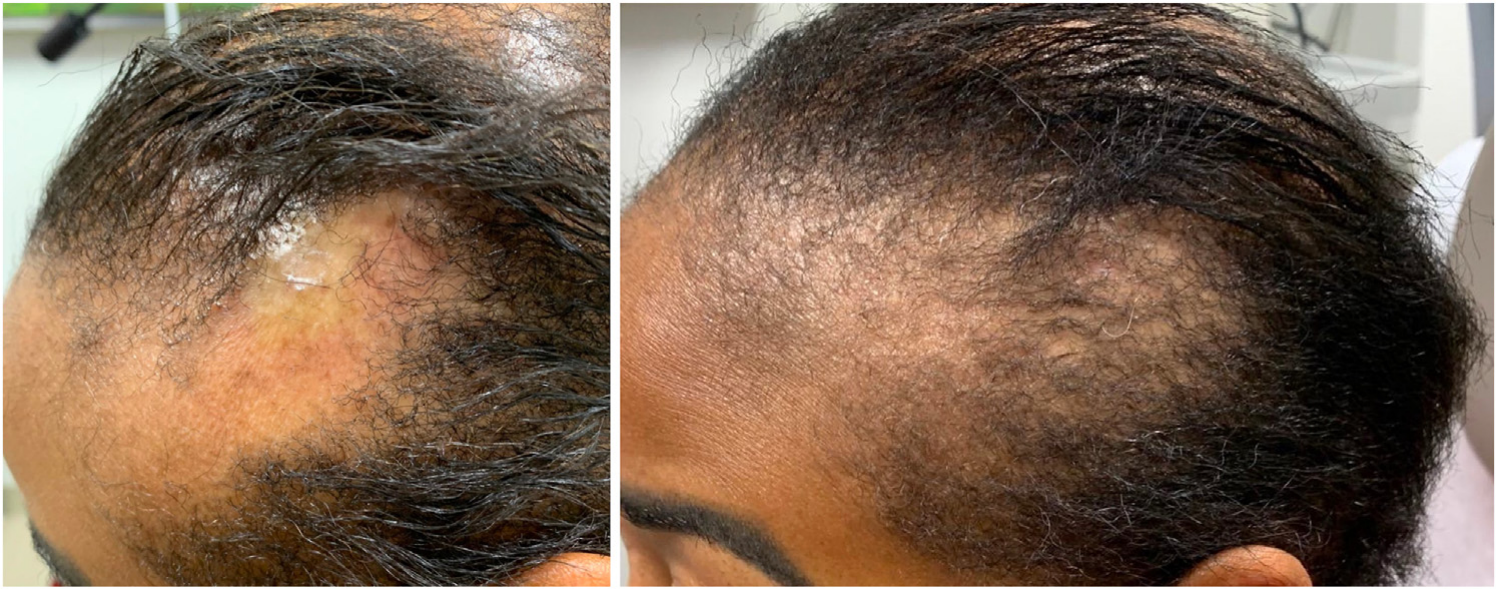
Alopecia areata before (*left*) and after (*right*) treatment with baricitinib.

On examination, the vertex scalp had significant alopecia with trichoscopy revealing loss of follicular ostia and perifollicular erythema. The parietal scalp was found to have discrete round patches of alopecia with exclamation point hairs and retained follicular ostia on trichoscopy (Figs 1 and 2). No other areas were affected and nail examination was not performed due to acrylic nails.

Two 4-mm punch biopsies were taken, one from the vertex scalp and the other from the left parietal scalp. The vertex scalp biopsy showed mucinous fibroplasia at the follicular infundibulum with associated lymphocytic infiltration, consistent with LPP. The biopsy taken from the left parietal scalp showed a reduced number of anagen hair follicles with follicular miniaturization and a marked increase in catagen follicles, consistent with AA.

She received intralesional triamcinolone injections to the vertex and bilateral frontal temples during this visit. She was also prescribed baricitinib 2 mg daily, doxycycline 100 mg twice daily for 1 month, and clobetasol 0.05% ointment for daily application to areas of hair loss.

She returned 3 months later reporting that she was doing well on baricitinib without any adverse effects. She reported not using clobetasol due to prior failed attempts. She denied new areas of hair loss and was noted to have noticeable scalp hair regrowth in both areas (Figs 1 and 2). Baricitinib was continued at the same dose as monotherapy.

## Discussion

In the presented case, the patient had simultaneous diagnoses of LPP and AA. This is a rare occurrence as there have been few reported cases.^4^ Both conditions individually pose diagnostic and therapeutic challenges, and their coexistence further complicates the management approach. The exact pathogenesis underlying the coexistence of these conditions is not well understood. A retrospective study of comorbidities in patients with LPP showed increased odds of AA.^5^ These data failed to achieve statistical significance; however, it may indicate the need to further elucidate whether there is a relationship between the 2 diseases. Similarly, both disease pathogeneses are thought to involve breakdown of the hair follicle immune privilege. Harries et al demonstrated that LPP is a result of loss of immune privilege at the hair follicle bulge leading to an increase in predominantly CD8 cytotoxic T cells and an accompanying increase in stem cell apoptosis in the hair follicle.^6^ The current theory for the pathogenesis of AA, on the other hand, suggests a loss of immune privilege at the hair bulb leading to a mixed inflammatory infiltrate including CD8 cytotoxic T cells, Th17 cells, dendritic cells, NK T cells, and mast cells.^7^ Loss of immune privilege at one part of the hair follicle may predispose loss at another site, which may explain the simultaneous existence of both diseases.^4^^,^^8^

Therapeutic recommendations for LPP typically include antimalarial drugs and corticosteroids (topical and intralesional). Treatment for limited AA includes topical and intralesional corticosteroids and minoxidil. Extensive involvement typically requires systemic corticosteroids, topical immunotherapy, or traditional immunosuppressants. More recently, Janus kinase inhibitors have been used as therapy for extensive, recalcitrant AA^2^ and have also shown to be promising in the treatment of LPP.^3^ Regarding the management of this complex case, the treatment regimen (intralesional corticosteroids, doxycycline, and baricitinib) was employed with the aim to suppress the autoimmune response, reduce inflammation, and promote hair regrowth. In this case, the patient received intralesional corticosteroids once and failed to use topical corticosteroids. She demonstrated a favorable response to treatment, displaying a reduction in inflammation, cessation of hair loss, and visible hair regrowth in all areas of hair loss, with the use of only baricitinib and a 1-month course of doxycycline. Additionally, Spencer et al^9^ highlight the limited efficacy of doxycycline in treating LPP, leading us to strongly believe the hair regrowth seen in this case can be attributed to baricitinib. This also further implicates the involvement of a similar autoimmune mechanism involving an aberrant immune response against hair follicles in both types of alopecia.

It is worth mentioning that the management of alopecia in Black or African American patients requires additional considerations. This case emphasizes the need for greater attention to the potential coexistence of multiple alopecias in this patient population, especially those with disease refractory to conventional treatments. Other types of alopecias, such as central centrifugal cicatricial alopecia, discoid lupus erythematosus, and traction alopecia, are more commonly observed in Black or African American patients and may occur concurrently potentially leading to misdiagnosis without careful clinical and histopathologic evaluation. Misdiagnosis could be compounded by differences in presentation due to intrinsic qualities of the hair and scalp seen in the skin of color patients.^10^ Thus, it is very important to use thorough trichoscopic evaluation in diagnosing alopecias in this patient population.

In conclusion, the presented case highlights the coexistence of AA and LPP in a Black or African American patient and therapeutic efficacy with treatment of both with baricitinib. Although complete hair regrowth was not achieved in this patient, baricitinib showed promising effects within the time period of use. Recognition of these conditions can be challenging, particularly in Black or African American patients due to the variety of alopecias that commonly affect this group; however, prompt diagnosis and management are necessary. More studies are needed for further insight into the pathogenesis of each disease to elucidate the cause of concurrent diseases highlighted in this case.

## Conflicts of interest

Dr Mayo has served as an investigator for Acelyrin, BMS, Galderma, Janssen, Lilly, and Pfizer. She has served as a consultant for Abbvie, Arcutis, BMS, Bodewell, Janssen, Lilly, Leo Novartis, Pfizer, and UCB. Drs Sanyi and Smith have no conflicts of interest to declare.
